# Strong light-matter coupling in van der Waals materials

**DOI:** 10.1038/s41377-024-01523-0

**Published:** 2024-08-21

**Authors:** Yuan Luo, Jiaxin Zhao, Antonio Fieramosca, Quanbing Guo, Haifeng Kang, Xiaoze Liu, Timothy C. H. Liew, Daniele Sanvitto, Zhiyuan An, Sanjib Ghosh, Ziyu Wang, Hongxing Xu, Qihua Xiong

**Affiliations:** 1 State Key Laboratory of Low-Dimensional Quantum Physics, Department of Physics, Tsinghua University, Beijing, 100084 China; 2 Division of Physics and Applied Physics, School of Physical and Mathematical Sciences, Nanyang Technological University, Singapore, Singapore; 3 CNR NANOTEC Institute of Nanotechnology, via Monteroni, Lecce, 73100 Italy; 4 Wuhan Institute of Quantum Technology, Wuhan, 430206 China; 5 School of Physics and Technology, Center for Nanoscience and Nanotechnology, and Key Laboratory of Artificial Micro- and Nano-structures of Ministry of Education, Wuhan University, Wuhan, 430072 China; 6 INFN National Institute of Nuclear Physics, Lecce, 73100 Italy; 7 Beijing Academy of Quantum Information Sciences, Beijing, 100193 China; 8 The Institute of Technological Sciences, Wuhan University, Wuhan, 430072 China; 9 Frontier Science Center for Quantum Information, Beijing, 100084 China; 10 Collaborative Innovation Center of Quantum Matter, Beijing, China

**Keywords:** Polaritons, Nanophotonics and plasmonics

## Abstract

In recent years, two-dimensional (2D) van der Waals materials have emerged as a focal point in materials research, drawing increasing attention due to their potential for isolating and synergistically combining diverse atomic layers. Atomically thin transition metal dichalcogenides (TMDs) are one of the most alluring van der Waals materials owing to their exceptional electronic and optical properties. The tightly bound excitons with giant oscillator strength render TMDs an ideal platform to investigate strong light-matter coupling when they are integrated with optical cavities, providing a wide range of possibilities for exploring novel polaritonic physics and devices. In this review, we focused on recent advances in TMD-based strong light-matter coupling. In the foremost position, we discuss the various optical structures strongly coupled to TMD materials, such as Fabry-Perot cavities, photonic crystals, and plasmonic nanocavities. We then present several intriguing properties and relevant device applications of TMD polaritons. In the end, we delineate promising future directions for the study of strong light-matter coupling in van der Waals materials.

## Abstract (web summary)

The review focuses on recent advances in TMD-based strong light-matter coupling, including the various optical structures strongly coupled to TMDs, several intriguing properties, and relevant device applications of TMD polaritons

## Graphical abstract

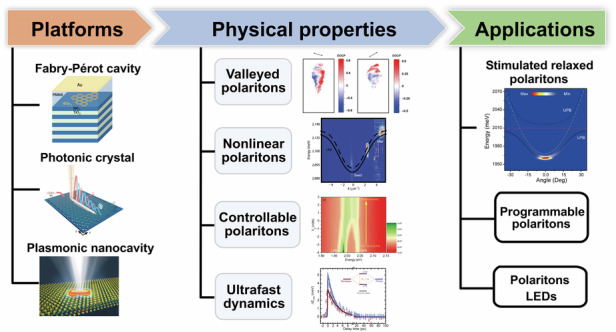

## Introduction

**Fig. 1:**
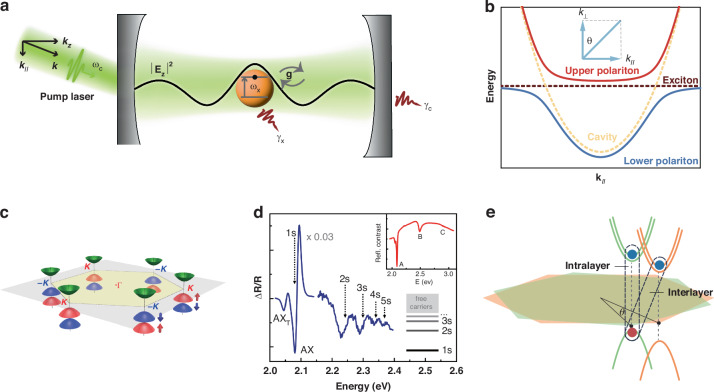
**Strong exciton-photon coupling and van der Waals materials.** **a** Schematic diagram of a cavity system with a single two-level active material embedded between two high-reflective mirrors. The parameters involved in the strong interaction regimes are the resonance frequency ${\omega }_{c}$ of the cavity mode, the resonance ${\omega }_{X}$ of optical gain materials, the light-matter interaction strength *g*, and the lifetime of the cavity photon ${\gamma }_{c}^{-1}$ and exciton ${\gamma }_{X}^{-1}$. **b** A typical exciton-polariton dispersion of a strongly coupled excitons and cavity mode. **c** The Brillouin zone and band structure at the *K* points. **d** The reflectance contrast derivative ($\varDelta R/R$) of a WS_2_ monolayer. The spectrum shows the ground state and the higher Rydberg-series states (2 s, 3 s, 4 s, 5 s…) of A exciton. **e** Schematic of a typical heterostructure formed by two different TMDs with twist angle $\theta$ in momentum space, resulting in an intralayer and interlayer exciton at the *K/K*’ valley. **c** Reprinted with permission from ref. ^27^ [American Physical Society]. **d** Adapted with permission from ref. ^32^ [American Physical Society]. **e** Reprinted with permission from ref. ^26^ [Springer Nature Limited]

The light-matter interaction lies in the heart of diverse optical processes, for instance, absorption and scattering of light, stimulated emission, and optical parametric amplification^1^. These optical processes can happen when the optical field does not significantly modify the dipole resonance in optical gain materials. Motivated by the exploration of uncharted physics territories and the imperative demands of quantum information science, enhancing and tailoring light-matter interaction have triggered a remarkable research endeavor recently. Microcavities (MCs), as optical resonators with sizes comparable to the wavelength of light, provide an effective way to confine photons, thus enhancing the light-matter interactions^2^. As the light field causes a perturbation on the electronic transition, the weak coupling is firstly satisfied, manifesting the widely known Purcell effect^3^. When the energy exchange between light and matter occurs more rapidly than their decay rates (Fig. 1a), this gives rise to the half-light, half-matter bosonic quasiparticles, referred to as microcavity exciton polaritons^4^. In particular, bound exciton states of semiconducting materials embedded into MCs can strongly couple with the cavity photons, leading to two new sets of eigenstates, which are referred to as the lower exciton-polariton branch and upper exciton-polariton branch with a finite vacuum Rabi splitting (Fig. 1b)^5^. Here, the exciton is regarded as an elementary excitation consisting of an electron and a hole bound together by Coulomb interactions^6^. Owing to the hybrid nature, exciton-polaritons inherit strong nonlinearities from their excitonic component and extremely small effective mass from their photonic component^7^. Based on these remarkable properties, microcavity exciton-polaritons have attracted tremendous attention, spanning from fundamental sciences, for instance, polariton superfluidity^8^, condensation^9^, quantum vortices^10^, and many others to practical device applications, such as low-threshold polariton lasers^11,12^, polariton spin transistor^13–15^, all-optical switches^16^ and so on. At early stages, exciton-polaritons have been studied mostly in semiconductor quantum-well systems, including GaAs-based^13^ and CdTe-based MCs^17^. However, the related exotic phenomenon, such as polariton condensation has been only demonstrated at cryogenic temperatures in GaAs and CdTe systems, limited by their small exciton binding energies. Thus, there has been a growing interest in searching for alternative systems to achieve room temperature polaritons. Over the past 15 years, ZnO^18^, GaN^19^, organic semiconductors^20^, and halide perovskites^11,21,22^ have appeared as promising candidates for bringing polariton physics to room temperature^5,23^.

More recently, due to their robust excitonic transition originating from the attractive Coulomb interaction and reduced screening in the low-dimensional environment, atomically thin transition-metal dichalcogenides (TMDs) have been an excellent candidate to support stable exciton-polaritons at ambient conditions^24^. The TMDs are a diverse family of materials with stable and well-explored 2H-phase MX_2_ compounds, where M represents a transition-metal element from group VI (M = Mo, W) and X is a chalcogen element (X = S, Se)^25^. Monolayer TMDs are direct bandgap semiconductors with the band extreme at the *K* and *K*’ points of the hexagonal Brillouin zone (Fig. 1d)^26–28^. Compared with other candidates on the frontier of room temperature polaritons, one of the most intriguing features of TMDs is the unique spin-valley locking^27^ at the *K* and *K*’ valleys, as a result of the strong spin-orbit interaction and the broken inversion symmetry. Thus, monolayer TMDs have two species of energy degenerate exciton states with opposite Berry curvature, which can be selectively excited by polarized light^29^, opening up new opportunities to achieve polarization-selective polaritonic devices^30,31^. In addition, the strong excitonic effect in monolayer TMDs introduces higher Rydberg-series excitonic states and charged exciton states (AX_T_)^32^, as shown in Fig. 1e. Furthermore, 2D van der Waals heterostructures made of different monolayer TMD materials have flexible electronic and optical properties relevant to their intralayer and interlayer exciton by controlling the twist angle (Fig. 1f)^25,26,33^, allowing unprecedented degree of freedom (DOF) to engineering the light-matter coupling in MCs. All of these unique properties signify that the emerging TMD system is a promising platform to enrich polaritonic physics and applications at room temperature.

Following the observation of TMD polaritons in 2015^24^, substantial progress has been made in the strong coupling of semiconductor TMDs to optical resonators. This progress encompasses the exploration of exotic polaritonic properties and the advancement of versatile polaritonic devices in TMD systems. In 2017, the investigation of valley-polarized exciton-polaritons in monolayer TMDs unveiled fresh avenues for the control and manipulation of coherent states of light and matter^31,34,35^. In the succeeding year, interacting polariton fluids^36^ and photonic-crystal exciton-polaritons^37^ have been successfully achieved within atomically thin TMDs, thereby broadening the range of possibilities for TMD polariton applications. In 2019, room temperature polariton light-emitting diodes (LEDs) in monolayer WS_2_ were reported, enabling the realistic polariton-based optoelectronic devices^38^. After a span of two years, significant progress was made in achieving the tunable exciton-polaritons through photonic lattices^39^, Moiré patterns^40^, and dielectric disorder^41,42^, revealing diverse engineering of the polariton confinement to control and shape the flow of TMD polaritons. Furthermore, polariton condensation has been reported in atomically thin TMD systems under both ambient conditions^43^ and cryogenic temperatures^44^ within the same year. In 2022, nonlinear phenomena of TMD polaritons have stirred growing research interest, including efforts to enhance nonlinear interaction strength^45–47^ and explore nonlinear polariton parametric emission^48^, which is the basis of the quest for novel polariton-based non-classical light sources and quantum simulators. Recently, Zhao et al.^49^ have explored the ultrafast dynamics of the nonlinear optical response in van der Waals superlattices strongly coupled to planar microcavities and directly observed the quenching of the Rabi splitting at ultrafast timescales.

This review not only aims to offer a broad overview of the current state of the TMD-based strong coupling but also attempts to inspire further investigations and developments of new polaritonic devices and technologies within van der Waals materials. For a detailed description of microcavity exciton-polariton physics and strong coupling with alternative promising materials, we refer the readers to some excellent reviews^5,23,50,51^. Here, we start with a brief introduction to TMD polaritons in various optical structures. Then, we present some basic properties of TMD polaritons, including the valley properties, nonlinearities, electrical-magnetic tuning, and transient dynamics. Moreover, we introduce recent polariton device applications based on the TMD system. In this section, stimulated relaxed/scattering polariton, programmable polaritons, and a polariton LED are reviewed. Finally, we anticipate potential research directions for studying exciton-polaritons based on van der Waals materials in the near future, encompassing nonlinear polaritonic simulators, quantum exciton-polaritons, Moiré exciton-polaritons, and anisotropic exciton-polaritons.

## TMD polaritons in various optical structures

**Fig. 2:**
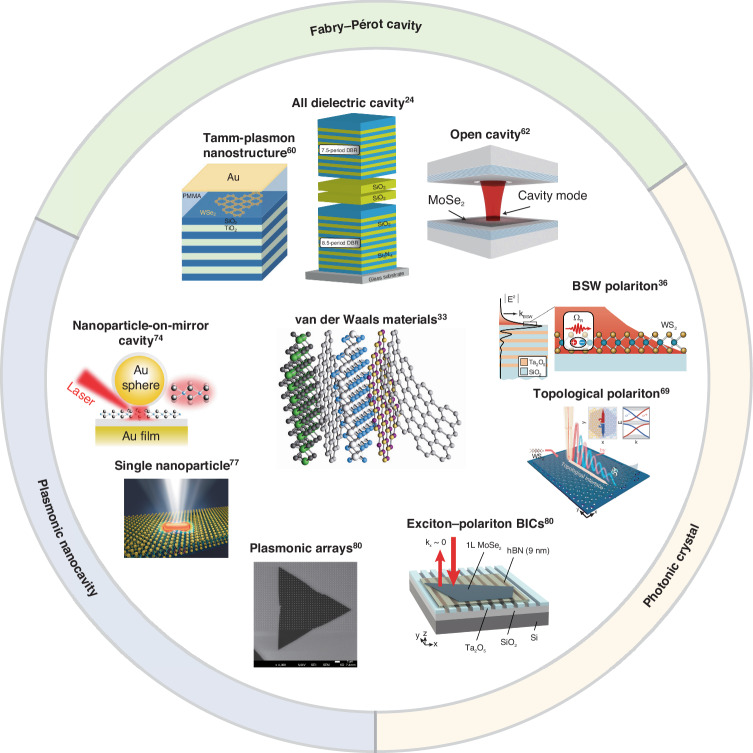
**The summary of diverse optical structures** for **TMD polaritons.** The cavities strongly coupled with 2D TMD semiconductors developed from the Fabry-Pérot cavity to other types of cavity structure, including plasmonic nanocavity and photonic crystal. Adapted with permission from ref. ^24,33,36,60,62,67,74^ [Springer Nature Limited]. Reprinted with permission from ref. ^69^ [AAAS]. Reprinted with permission from ref. ^77,80^ [American Chemical Society]

Thanks to the improved semiconductor fabrication capabilities, the tightly bound excitons in TMD materials can strongly couple with the diverse types of photon modes that are confined in different optical cavities and nanostructures. Figure 2 briefly enumerates typical works about TMD polaritons in various optical structures, including Fabry-Pérot cavities^24,52,53^, photonic crystals (PCs)^36,37,54^, and plasmonic nanocavities^55–57^.

The majority of optical resonators designed for strong coupling with two-dimensional semiconductors are based on the Fabry-Pérot (FP) cavity, which sandwiches the optical active material, for instance, monolayer and multilayer TMDs, and TMD heterostructures. In 2015, the first realization of TMD exciton-polaritons was reported by Liu et al. in an all-dielectric FP microcavity at room temperature^24^. Their resonator structure consisted of a monolayer of MoS_2_ embedded between two highly reflecting distributing Bragg reflectors (DBR), which were designed by dielectric layers of alternatively high and low refraction indices with an optical thickness of $\lambda /4$ per layer and grown by plasma-enhanced chemical vapor deposition. By angle-resolved reflectivity and photoluminescence maps, they observed a Rabi spitting of $46\pm 3{\rm{meV}}$, demonstrating that the FP cavity is an effective means of achieving strong coupling in TMD systems. In subsequent developments, there are three kinds of optimization methods for DBR-based microcavities. The first one is to enhance the quality factor, which can be up to 11,000 by using a transferrable DBR^58,59^. Then, a smaller mode volume is also pursued to manifest strong coupling conditions at room temperature. In 2016, to reduce the mode volume, Lundt et al. implemented a Tamm-plasmon cavity by replacing the top DBR with a 35 nm-thick gold layer^60^. Using this structure, they unequivocally unveiled the emergence of exciton-polaritons in monolayer WSe_2_ cavities, a phenomenon hitherto unobserved in these types of 2D TMD materials, even at cryogenic temperatures. Due to the high absorption induced by the metal mirror, their microcavity structure sacrificed the quality factor^61^. What’s more, the structure consisting of two silver mirrors sandwiching the 2D WS_2_ layer has also been reported, showing a Rabi splitting up to 100 meV^34^. Finally, to flexibly tune the cavity resonance and lower the local strain induced in the process of sample fabrication, an open cavity by mounting the top mirror on a motorized piezo also developed a lot in TMD systems^62–64^.

Recently, dielectric PCs have been designed to strongly couple with 2D TMD systems for their optical field confinement and high-quality factor. The simplest one-dimensional (1D) PC structure is the Bragg mirror. At the air-dielectric interface of a Bragg mirror, the Bloch surface waves (BSW) can be supported by propagating over hundreds of micrometers^65^. Strongly coupling a monolayer TMD to a propagating BSW gives rise to the formation of exciton-BSW polaritons (BSWPs), inheriting both long-range propagation and strong nonlinearities^36,66^. Another type of 1D PC is the dielectric grating, where a 2D semiconductor can be directly placed on it. In 2018, Zhang et al. first combined TMDs with an anisotropic in-plane grating in the strong coupling regime at room temperature and observed highly anisotropic dispersions^37^. However, more elaborate PCs will facilitate studying complex and exotic polaritonic physics based on TMD materials. For example, optical bound states in the continuum (BICs) are distinctive photonic modes confined within the continuum spectrum, supported by PC structures with certain geometries. These states find applications in nanophotonics, enabling precise light manipulation, and hold promise in areas like sensors and lasers. In 2020, optical BIC-based polaritons were first reported by transferring an atomically thin semiconductor MoSe_2_ onto a PC slab, showing a Rabi splitting of 27 meV^67^. An alternative solution is to engineer nontrivial PCs with topologically protected edge or surface states where polaritons are topologically protected from back-scattering, and their helical propagating can be unambiguously demonstrated^68,69^. Compared with bare photonic systems, topological polaritons foster the growth of actively controllable topological devices with giant optical nonlinearity and enhanced electromagnetic tunability. Another recent work about polariton devices based on topological protection involves the strong coupling of a WS_2_ monolayer with topologically protected BIC resonance in the BSW supported by DBR^70^. In this configuration, through the exploitation of the strong enhancement of the BSW field and precise engineering of the patterned surface, they achieved maximum coupling efficiency with minimal modal losses, utilizing the topological protection provided by the BIC state. As a result, it was possible to attain the highest cooperativity (defined as $\frac{4{g}^{2}}{{\varGamma }_{exc}{\varGamma }_{ph}}$), which is far more than 37 for a strongly coupled TMD. Here, *g* is the light-matter interaction constant, and *Γ*_*exc*, *ph*_ denotes the measured linewidths for neutral exciton and the unloaded photonic mode, respectively.

To further reduce the mode volume, plasmonic nanocavities have attracted increasing attention since they are capable of overcoming the optical diffraction limit. Figure 2 summarizes some remarkable works on TMD-based plasmonic nanocavities, including the strong plasmon-exciton coupling within nanoparticle-over-mirror resonators^71–75^, individual nanoparticles^76–79^, and plasmonic arrays^80,81^. Though having low-quality factors, plasmonic nanocavities are promising candidates for further increasing the Rabi splitting, as a result of their ultrasmall mode volume^82^. For example, the strong coupling of an MoS_2_ monolayer in an assembled gold nano resonator with an Al_2_O_3_ spacer realized a Rabi splitting up to 130 meV^74^. Moreover, by placing a single silver nanorod on a monolayer TMD, Zheng et al. observed the formation of hybrid plexcitonic states and achieved a Rabi splitting as large as 49.5 meV, approaching but not definitively meeting the criteria for strong coupling^78^. This result also underscores the considerable challenge of achieving strong coupling in plasmonic nanocavities, namely balancing the increased Rabi splitting with the reduced quality factor. In the same year, another study reported the strong plasmon–exciton coupling in an Au nanorod-WS_2_ hybrid system and demonstrated the active control of the light-matter coupling by applying the external gating and scanning temperature^77^. For plasmonic arrays, in 2016, Liu et al. first demonstrated the strong exciton-plasmon coupling in a silver nanodisk lattice pattered on the monolayer MoS_2_, where three types of resonances were hybridized, including an exciton, a plasmonic lattice mode, and a localized surface plasmon (LSP) mode^80^. Such a coupling showed pronounced splitting up to 58 meV and can survive at room temperature. In the same year, Ebbesen and co-workers studied the coupling of WS_2_ monolayer to plasmonic arrays and reported a Rabi splitting of 60 meV under the strong coupling regime at room temperature^81^. Beyond coupling strength, another significant advantage of reducing the mode volume with plasmonic nanocavities is the decrease in the number of TMD excitons involved in the strong coupling. When the number of excitons contributing to the coupling is diminished to the level of a few excitons (<10), plasmonic systems have the potential to meet the requirements of quantum optics, serving as the foundation for single-photon sources and quantum information processing. This is exactly the motivation behind the recent exciting progress in few-excition strong coupling in plasmonic nanocavity involved TMD active gain materials^72,73,75^. However, it is quite challenging to reach the few-exciton regime, as recently debated on this controversy^83^.

To date, the aforementioned three optical structures have emerged as the principal platforms for achieving strong coupling. Nevertheless, TMDCs in flake form, when possessing adequate thickness, have the capability to manifest Fabry-Pérot-type resonances by themselves, thereby forming self-hybridized exciton-polariton without reliance on an external resonator^84–86^. To facilitate a comprehensive comparison of the distinctions among these optical structures, Table 1 summarizes the Rabi splitting and operating temperature of exciton polaritons from the earliest studies demonstrating the strong couplings, along with valley phenomena, nonlinear interaction, propagation, and spatial coherence in various platforms.

**Table 1:** Comparison of Rabi splitting and operating temperature of exciton polariton based on different TMD materials

Platform	Materials	Description	Quality factor/photonic linewidths	Rabi splitting (meV)	Research content^Ref.^
Fabry-Pérot cavity	WS_2_	DBR + DBR	400	40 (monolayer, 110 K)	Formation of exciton polariton^52^
			2000	37 (monolayer, RT)	Polariton condensate^43^
				42 (monolayer, 20 K)	Polariton parametric emission^48^
			3000	25 (monolayer, RT)	Polariton propagation^42^
		DBR+Ag	120	80 (monolayer, RT)	Macroscopic valley-polarized polaritons^35^
		Ag+Ag	25–30	70–100 (monolayer, RT)	Optical control of valley polaritons^34^
	WSe_2_	DBR+metal	110	23.5 (monolayer, RT)	Formation of Tamm-plasmon-exciton polariton^60^
			220	7.7 (multiple layers, 15 K, 2 s exciton state)	Enhanced nonlinear interaction of Rydberg polaritons^47^
		DBR + DBR		~26 (monolayer, 4.2 K)	Valley coherent exciton-polaritons^30^
			240	11.4 (monolayer, RT)	Room temperature valley coherence^53^
			4300 ± 400	30 (monolayer, RT)	Spatial coherence of exciton polariton^41^
	MoSe_2_	DBR + DBR	4600	46 (monolayer, 5 K)	Optical Valley Hall effect^59^
		Open cavity	~850	~4.4 (monolayer, 50 K, trion)	Valley-addressable exciton/trion- polaritons^62^
				20 (monolayer, 4.2 K)	Exciton-polaritons in van der Waals heterostructures^64^
	MoS_2_	DBR + DBR	ħΓ_cav_ = 9 meV	46 ± 3 (monolayer, RT)	Formation of exciton polariton^24^
			ħΓ_cav_ = 10 meV	39 ± 5 (monolayer, RT)	Valley-polarized exciton-polaritons^31^
		DBR+Ag	130	54 (monolayer, RT)	Formation of Tamm-plasmon exciton-polaritons^61^
Photonic crystal	WS_2_	BSW	924 (at 650 nm) 795 (at 645 nm)	43 (monolayer, RT)	Interacting polariton fluids^36^
		1D PC	ħΓ_cav_ = 6.5 meV	22.2 (monolayer, RT)	Photonic-crystal exciton-polaritons^37^
		Topological polariton	ħΓ_cav_ = 6.5 meV	38 ± 1 (monolayer, 160 K)	Generation of helical topological exciton-polaritons^69^
		BIC-BSW	ħΓ_cav_ << 4.5 meV	>70 (monolayer, RT)	Strongly enhanced light-matter coupling^70^
	WSe_2_	Metasurface	143	18 (monolayer, RT)	Metasurface integrated exciton-polaritons^54^
		BSW	641	56.8 (monolayer, RT) 111.13 (4 L, RT)	Boosting exciton transport^66^
	MoSe_2_	BIC	>900	27 (monolayer, RT)	The formation of BIC-like polaritons^67^
Plasmonic Nanocavity	WS_2_	Plasmonic arrays	ħΓ_cav_ = 247.8 meV	138 (monolayer, RT)	Formation of strong plasmon–exciton couplings^55^
		Single nanoparticle	ħΓ_cav_ = 130 meV	150 (monolayer, 6 K, trion)	Tunable charged exciton polaritons^76^
			ħΓ_cav_ = 149 meV	91–133 (monolayer, RT)	Formation of strong plasmon–exciton couplings^77^
		Nanoparticle-on-mirror	ħΓ_cav_ = 180 meV	~163 (monolayer, RT)	Revealing Strong Plasmon-Exciton Coupling^73^
			ħΓ_cav_ = 220 meV	~240 (monolayer, RT)	Greatly Enhanced Plasmon-Exciton Coupling^56^
	MoS_2_	Nanoparticle-on-mirror	ħΓ_cav_ = 45 meV	~130 (monolayer, RT)	Nonlinear valley phonon scattering^74^
			ħΓ_cav_ = 280 meV	190 (monolayer, RT)	Manipulating coherent light-matter interaction^57^
		Plasmonic arrays	ħΓ_cav_ = 90–116 meV	58 (monolayer, 77 K)	Formation of strong plasmon–exciton couplings^80^
	WSe_2_	Nanoparticle-on-mirror	ħΓ_cav_ ≈ 66 meV	>135 (multiple layers, RT)	Formation of strong plasmon–exciton couplings^75^
		Single nanoparticle	ħΓ_cav_ = 110 meV	~100 (multiple layers, RT)	Formation of strong plasmon–exciton couplings^79^
Self-hybridized cavities	WS_2_	Bulk TMDCs on a glass substrate		~235 (thickness > 60 nm, RT)	Formation of self-hybridized exciton-polaritons^84^
		Patterned bulk TMDCs	ħΓ_grating_ = 133 meV ħΓ_exciton-cavity_ = 50 meV	410 (multiple layers, RT)	Nanopatterned multilayer WS_2_ grating resonators^86^
			ħΓ_cav_ = 30 meV	~116 (thickness = 45 nm, RT)	BIC-driven intrinsic strong coupling^85^

*RT* room temperature, *ħΓ*_*cav*_ the linewidth of a cavity mode, *ħΓ*_*grating*_ the linewidth of the dielectric gating mode, *ħΓ*_*exciton-cavity*_ the linewidth of the exciton-cavity hybrid mode

## Basic properties in the TMD polariton system

With the realization of strong coupling between TMD materials and diverse optical structures, a growing range of distinctive physical properties within the TMD polariton system have been unveiled. In this section, we will review valley properties, nonlinearities, electrical-magnetic tuning, and transient dynamics of TMD polaritons.

### Valley phenomena in TMD microcavities

**Fig. 3:**
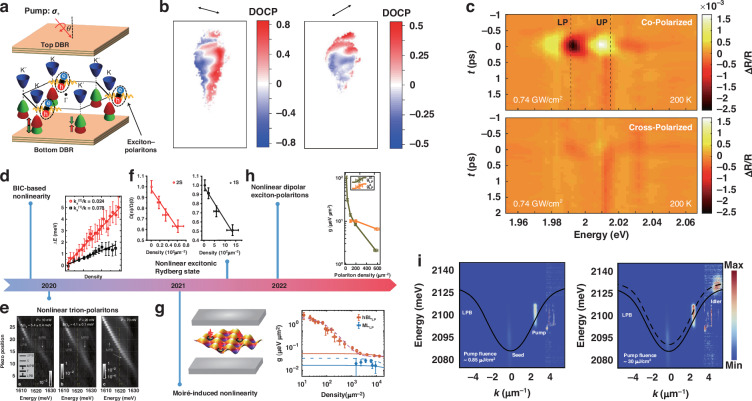
**Valley properties and nonlinearities of TMD polaritons.** **a** The schematic of valley polaritons in a TMD microcavity. The circularly polarized light (${\sigma }^{+}$) primarily excites exciton-polaritons in the *K* valleys due to the spin-valley locking of monolayer TMDs. **b** The spatially resolved DOCP distribution across the monolayer with different polarization orientations of the excitation light evidences the optical valley Hall effect. **c** Pump-induced differential reflectance ($\varDelta R/R$) when pump and probe are co-polarized (upper panel) and cross-polarized (lower panel). **d** Nonlinear interaction of BIC-based polaritons. The black squares and red circles, respectively, represent the spectral blueshift of the lower polariton branch from ${k}_{x}^{(1)}/k=0.078$ and ${k}_{x}^{(2)}/k=0.024$, with their corresponding fitting curves (black and red lines). **e** Nonlinear quench of trion-polariton splitting for different pump powers: 10 nW, 20 nW, and 70 nW, respectively. **f** Normalized Rabi splitting versus the polariton density for 2 s and 1 s exciton-polaritons. **g** Moiré-induced polariton nonlinearity. The left panel is the schematic of the Moiré polariton system and the right panel shows the nonlinear coefficient as a function of polariton density for the Moiré systems (red) and the monolayer systems (blue). **h** Nonlinear coefficient versus the polariton density from the dipolar exciton-polaritons and A exciton-polaritons. **i** Nonlinear polariton parametric emission from monolayer WS_2_ MCs. The idler is not visible at low pump fluence (left panel) but visible at high pump fluence (right panel). **a** Reprinted with permission from ref. ^31^ [Springer Nature Limited]. **b** Reprinted with permission from ref. ^59^ [Springer Nature Limited]. **c** Reprinted with permission from ref. ^94^ [Springer Nature Limited]. **d** Adapted with permission from ref. ^67^ [Springer Nature Limited]. **e** Reprinted with permission from ref. ^46^ [Springer Nature Limited]. **f** Reprinted with permission from ref. ^47^ [Springer Nature Limited]. **g** Reprinted with permission from ref. ^40^ [Springer Nature Limited]. **h** Reprinted with permission from ref. ^45^ [Springer Nature Limited]. **i** Adapted with permission from ref. ^48^ [Springer Nature Limited]

Exciton polaritons in 2D TMDs possess valley polarization from their exciton constituents, thereby revealing control of a new DOF and unique dynamics that were not available in previous semiconductors. 2D exciton polaritons in TMDs demonstrated (Fig. 3a) that their circular polarization is well preserved when compared to bare excitons^31^. This preservation of circular polarization is feasible at elevated temperatures (even at room temperature)^34^. Similar circular polarization dependence was also observed in trion polaritons under electrical doping^30,62,87^. The observations of valley polarization in 2D exciton-polaritons can be attributed to the coherent coupling processes that suppress valley exciton depolarization processes of intervalley scattering and dephasing.

These observations trigger in-depth exploration of the valley DOF for valleytronic and polaritonic applications, including the emerging topics of valley coherence and the valley Hall effect. Valley coherence is a dynamic process that was initially observed in bare 2D TMD excitons^88,89^ in terms of their linear polarization dependence. The angle of linear polarization reflects the correlated phase between the *K* and *K*’ valleys because the linear polarization of light can be decomposed as a superposition of two cross-circularly polarization light components. When the linear polarization of exciton photoluminescence maintains the same angle as the pumping light, the correlated phase between two valleys does not change and is thus identified as coherent valley dynamics. However, it is challenging to observe valley coherence in bare valley excitons at elevated temperatures, due to a typical valley coherence time of a few picoseconds and a complex decoherence process^90,91^. The decoherence process generally involves a competition between population relaxation, valley scatterings, and pure dephasing^8^. When the population relaxation dominates, excitons maintain valley coherence and their emission shows the same linear polarization with the pump light. Conversely, if the valley scattering and dephasing dominate, the valley coherence cannot be observed in the linear polarization dependence of excitonic emission. Nevertheless, polaritons could exhibit and even enhance valley coherence^30^ up to room temperature^35,53^. The valley coherence of 2D exciton-polaritons is supported by the coherent strong coupling process, which protects valley coherence from dephasing at high temperatures. For the valley Hall effect, excitons or carriers from different valleys drift in opposite directions, which are both perpendicular to their transport pathways. The valley Hall effect was initially predicted in graphene and ultimately achieved in MoS_2_ transistors^92,93^, considered a crucial mechanism for valleytronics. This phenomenon has also been recently reported in 2D exciton polaritons^59^, in which the valley coherence is maintained through second harmonic generation. The distribution of the degree of circular polarization (DOCP) in monolayer MoSe_2_ reveals two domains separated by cross-circular polarization emissions, and the DOCP distribution undergoes significant changes under different linear polarization angles (Fig. 3b). In light of valley DOF, intriguing manipulations of valley-polarized polaritons under external fields such as ultrafast light, magnetic fields, and electric fields have also been investigated. One notable work is the observation of the valley-selective Stark effect in an FP cavity integrated with monolayer WS_2_^94^. In pump-probe configuration, Stark shifts are only evident when the pump and probe light are co-circular polarization (Fig. 3c).

### Nonlinear interaction of TMD polaritons

Exciton-polaritons, characterized as half-light and half-matter quasiparticles, provide a fascinating platform for studying nonlinear optical phenomena at relatively low excitation density, especially in comparison with pure photonics systems. Investigating the nonlinearities inherent to polaritons holds significance in elucidating the underlying nonlinear optical processes, which form the basis of the polariton devices. As for the TMD polaritons, a seminal contribution by Barachati et al. marked a significant milestone by demonstrating the existence of polariton interactions through the strong coupling between monolayer WS_2_ and BSWs^36^. This is evidenced by the formation of upper and lower polariton branches, along with a clear anti-crossing feature in the reflectivity spectra. The polariton-polariton interactions have been studied by employing pulsed resonant excitation of the lower polariton in a reflection configuration, which revealed clear blueshifts with increasing polariton density. The interaction strength can be estimated from the experimental blueshift of lower polariton, a crucial quantitative metric of these phenomena. Recent advancements in the field have introduced optical BICs as a novel approach for engineering resonances within PCs. Kravtsov et al. investigated nonlinear polaritons arising from the strong coupling of excitons within monolayer the cavity photonic mode at low temperatures^67^. The narrow line widths of the polariton modes enable an insightful measurement of polariton-polariton interaction strengths, as discerned through power-dependent blueshifts observed in resonant reflectance measurements. As shown in Fig. 3d, the blueshift values for different *k* extracted from Fano line shape fitting, demonstrate a linear correlation with increasing fluence. Furthermore, the exciton-exciton interaction strength can be estimated *via* the polariton-polariton interaction strength, accounting for the Hopfield coefficient, which is in the same order as the theoretical estimation. Then, combining the flexible tuneability of a topologically protected BIC resonance in the BSW mode, Maggiolini et al. observed the enhancement of the strong light-matter interactions and large polariton nonlinearities, even at room temperature, signifying promising prospects for the development of practical polariton devices^70^.

Subsequent research endeavors have extended this exploration by employing planar microcavities to investigate the nature of the polariton interactions within TMD monolayers. By utilizing a MoSe_2_ monolayer within a microcavity, Stepanov et al. examined the blueshift of the lower polariton branch, using spatially resolved optical transmission spectroscopy facilitated by pulsed laser excitation^95^. The quantification of optical nonlinearities from the fermionic saturation and Coulomb interaction between excitons has been determined by polarization-dependent nonlinear transmission measurements. When compared with the realistic values expected in the hydrogenic picture, an enhancement in both excitonic fermionic saturation and exciton-exciton interactions has been observed, which offers new perspectives for harnessing exciton-mediated optical nonlinearities.

Moreover, the presence of neutral excitons is known to be strongly bound, resulting in comparatively reduced nonlinear optical effects when compared to the previous system involving gallium arsenide (GaAs). Since the nonlinear coefficient is proportional to the overlap between the wave functions of particles^96^, a common approach to enhance the nonlinear optical effects is to form polaritons with higher-order excitations. Tan et al. conducted a notable investigation involving the introduction of itinerant electrons into monolayer molybdenum (MoSe_2_), wherein they observed the emergence of polaron-polaritons exhibiting enhanced interactions^97^. In comparison to exciton-polaritons, the polaron-polaritons exhibited an enhancement factor of approximately 50, as ascertained through time-resolved pump-probe experiments. Furthermore, Emmanuele et al. reported on the strong coupling of trion states within monolayer MoSe_2_, characterized by low electron densities, with a photonic mode in a planar microcavity^46^. Remarkably, even with relatively low photon fluence, trion polaritons exhibited significant energy shifts attributable to phase space-filling effects. Figure 3e illustrates the strong coupling between trion and cavity modes, evidenced by an anti-crossing phenomenon at the lowest pulse power of 10 nW. As the pump power increased, the Rabi splitting diminished, eventually reaching a regime of total saturation at 70 nW. In contrast to neutral exciton-polaritons, trion polaritons demonstrated an interaction strength enhancement in the range of 10 to 100. Moreover, in the context of excited-state exciton exhibiting a larger Bohr radius, when coupled with a monolithic microcavity, the emergence of 2 s polaritons has been characterized by higher nonlinearities^47^. Figure 3f shows the density-dependent Rabi splitting for the excited 2 s and 1 s excitons. It is noteworthy that the interaction strength associated with 2 s polaritons surpasses that of 1 s polaritons by a factor of ~4.6, which shows a good agreement with the scaling principles governing Bohr radius-related phenomena.

Moiré excitons, which are achieved through slight lattice or crystal orientation mismatches in stacked monolayers, offer a promising avenue for enhancing the nonlinearities. Coupling these excitons with a planar microcavity, Zhang et al. pioneered the observation of Moiré exciton polaritons^40^. As shown in Fig. 3g, the measured nonlinear coefficient for Moiré polaritons surpassed that of monolayer polaritons, exhibiting remarkably high nonlinearities at exceedingly low densities, attributed to exciton blockade within each Moiré cell. Additionally, the nonlinearities can be enhanced by the coupling of microcavity modes and excitonic states featuring physically separated electrons and holes. For example, interlayer excitons, characterized by significant out-of-plane electric dipole moments, offer an intriguing avenue. Two independent research groups separately reported the strong coupling of microcavity photons with interlayer excitons in bilayer MoS_2_^45,98^. In contrast to exciton polaritons within a monolayer, they observed approximately a tenfold increase in nonlinearities in di-polaritons (Fig. 3h). These enhanced nonlinearities show the potential to push the TMD polariton system into the quantum nonlinear regime.

Besides enhancing nonlinearities, a crucial challenge lies in the observation of prominent nonlinear effects, which form the fundamental basis for nonlinear devices. Recently, a development involving nonlinear optical parametric polariton scattering has been reported within a planar microcavity based on monolayer WS_2_^48^. As shown in Fig. 3i, under pulsed resonant excitation at the inflection point, triggering the ground state, the emergence of an idler state at high *k* serves as clear evidence of the nonlinear process. This process was accompanied by amplified transmitted trigger signals, linewidth narrowing, and energy blueshift, all of which were discernible even at room temperature. Additionally, through the resonant injection of the dispersion at specific wave vectors, nonlinear self-amplification of polariton emission for valley-dependent ground states has been reported by Cilibrizzi et al.^99^.

### Controlling polaritonic properties by electrical-magnetic tuning

**Fig. 4:**
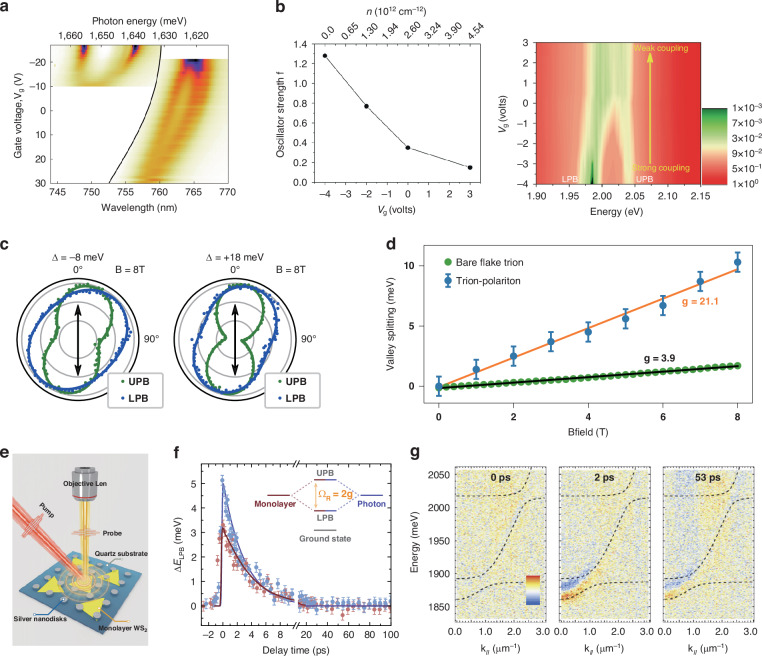
**Electromagnetic field tuning and transient dynamics of TMD polaritons.** **a** The transmission spectrum of an open cavity is strongly coupled with monolayer MoSe_2_ as a function of the gate voltage (vertical scale) that allows for varying the electron density. The left (right) part is the transmission when the open cavity is tuned to resonance with the repulsive (attractive) polaron. **b** Controllable strong light-matter interaction strength in monolayer WS_2_ MCs through electric field gating (the right panel). The left panel shows the oscillator strength as a function of the gate voltage, which determines the carrier concentration. **c** Polariton intensity versus the linear polarization angle in detection under a magnetic field with different cavity detuning. **d** Giant effective trion-polariton Zeeman splitting. The g-factors of the trion-polariton and bare trion are 21.1 ± 0.9 and 3.93 ± 0.04, respectively. **e** The schematic illustration of time-resolved pump-probe microscopy to study the ultrafast optical responses in the Ag nanodisk (ND)-WS_2_ hybrid system. **f** The blueshift of the lower polariton branch versus the time delay for low (red) and high (blue) pumping fluence, illustrating a single exponential decay for the monolayer MC. **g** The angle-resolved transient reflectivity spectroscopy mapping of het@cavity. **a** Reprinted with permission from ref. ^100^ [Springer Nature Limited]. **b** Reprinted with permission from ref. ^101^ [American Chemical Society]. **c** Reprinted with permission from ref. ^30^ [Springer Nature Limited]. **d** Reprinted with permission from ref. ^87^ [Springer Nature Limited]. **e** Reprinted with permission from ref. ^104^ [Springer Nature Limited]. **f** Reprinted with permission from ref. ^49^ [Springer Nature Limited]. **g** Reprinted with permission from ref. ^107^ [Springer Nature Limited]

Compared with quantum wells or 2D electron systems in III–V semiconductors, monolayer TMDs exhibit strong Coulomb interaction inheriting from their lowered dimensionality, the reduced dielectric screening, and the relatively large carrier effective masses^32^. Thus, TMD systems open up new opportunities to investigate and engineer many-body physics, such as Bose-Fermi mixtures by electrostatic control in optical cavities. In 2016, Sidler et al. first carried out an investigation of Fermi polarons in a fiber cavity strongly coupled with MoSe_2_ monolayer at low temperature^100^. Figure 4a shows that when the electron density is introduced by increasing gate voltage, the normal mode splitting for the repulsive (attractive) polaron tends to decrease (increase). These results indicate that the oscillation strength is gradually transferred from the higher-energy repulsive polaron resonance to the lower-energy attractive polaron resonance. However, room temperature control of the light-matter coupling strength has always been highly sought after for realistic devices such as polaritonic modulators, switches, and logic elements. In the following two years, Chakraborty et al.^101^ created a device consisting of a WS_2_ field effect transistor inside a metal mirror-cavity structure. When free carriers were pushed into monolayer WS_2_ under gating, a significant reduction of the exciton oscillator strength was demonstrated (the left panel in Fig. 4b), resulting in the transitions from strong to weak coupling at room temperature (the right panel of Fig. 4b). In addition, gating detuning has also been realized in other types of coupling system. For example, Lee et al.^102^ presented electrical tuning of the exciton-plasmon interaction in monolayer MoS_2_ integrated with plasmonic nanoresonators.

The versatile control of the internal degrees of freedom of electrons is regarded as the heart of condensed matter physics. Due to the spin-valley locking in 2D TMDs, the valley pseudospin is allowed to be directly manipulated by external magnetic fields, similar to electron spin^88^. Therefore, compared with other materials for room temperature polaritonics, magnetic tuning of exciton-polaritons in TMDs has more potential to explore and tune exotic pseudospin physics. In 2018, by applying an out-of-plane magnetic field to a monolayer WSe_2_ placed in an open microcavity, Dufferwiel et al. manipulated the valley coherent exciton-polaritons induced by the precession of the pseudospin vector^30^. By collecting PL intensity under different detection angles, they observed a detuning-dependent rotation of the linear polarization plane under linearly polarized excitation (Fig. 4c). More interestingly, their rotation angles were up to three times larger than the bare exciton under the same magnetic field strength. Another fascinating work is the observation of a giant effective trion-polariton Zeeman splitting, over five times larger than the bare flake trion splitting (Fig. 4d) because of spin-selective strong light-matter coupling^87^. In stark contrast, the magnetic field-induced splitting for exciton-polaritons was in line with the splitting of bare excitons, which is revealed in the work by Lundt et al.^103^. Furthermore, the magneto-optical response was also focused on trapped exciton-polariton systems, displaying an analogous Zeeman energy-splitting^41^.

### Ultrafast dynamics of polaritons in TMD microcavities

Although the previous steady-state measurements have undoubtedly confirmed the existence of strong coupling between TMDs and microcavities, present static approaches encounter challenges in having a comprehensive understanding of these hybrid light-matter states, particularly from a transient temporal perspective. Figure 4e illustrates the experimental setup for time-resolved pump-probe microscopy, presenting a new way to explore ultrafast optical responses in *k*-space and real-space measurements of TMD microcavities^104^. In 2019, Tang et al. achieved coherent strong coupling between a PC slab and a monolayer WS_2_^105^. This pioneering work delved into the ultrafast dynamics of both WS_2_ and WS_2_-PhC polaritons through off- and near-resonance excitations. Their investigations unveiled the significant influence of nonequilibrium thermal decay-induced Coulombic screening on the formation of exciton-polaritons. Additionally, Du et al. studied the exciton-plasmon coupling within a nanodisk hybrid system in ultrafast time domain^106^. Following femtosecond laser excitation, the appearance of photoinduced absorption signals at the Fano resonance frequency has been observed, accompanied by neighboring bleaching signals due to rapid reductions in exciton-plasmon coupling. Then, a quick recovery of the Fano resonance with a sub-100 fs time scale has been observed, due to the transfer of energy from excitons to plasmons. It illustrated that the intrinsic carrier relaxations take place on the picosecond time scale. In 2024, Hu et al. elucidated the polariton relaxation dynamics within an FP microcavity strongly coupled with the hBN/MoS_2_/hBN/WS_2_ heterojunction (het@cavity). As shown in Fig. 4g, the *k*-space time-resolved spectroscopy reveals that the polariton population at *k*_//_ = 0 μm^−1^ from het@cavity reaches the maximum at ~2 ps, exhibiting a significantly faster polariton relaxation compared to a WS_2_ microcavity. This accelerated polariton relaxation arises from markedly enhanced intra- and inter-branch exciton-exciton scattering, effectively overcoming the hot phonon bottleneck effect^107^.

Furthermore, the utilization of time-resolved pump-probe techniques has emerged as a powerful tool for exploring polariton nonlinearities. In 2022, Tang et al.^104^ observed substantial room temperature nonlinearities in plasmon-exciton polaritons, a hybrid system comprising a monolayer of WS_2_ and silver nano-disks. Their ultrafast pump-probe experiments demonstrated that the plexciton nonlinearities are primarily driven by saturation and higher-order exciton-induced dephasing interactions, thereby offering a promising avenue for manipulating the nonlinear absorption properties of the solid-state system within ultrafast time domains. In another noteworthy endeavor, recently multiple WS_2_ monolayers have been introduced into a planar microcavity, yielding enhanced Rabi splitting in the superlattice microcavity^49^. Employing a pump-probe configuration, the authors investigated the temporal dynamics of nonlinearities, revealing their sensitivity to the existence of dark excitations. As shown in Fig. 4f, the presence of long-lived dark excitation has been observed within the superlattice microcavity.

## Polaritonic device applications

As an escalating number of polariton properties are unveiled, scholarly attention has progressively pivoted towards the exploration of polaritonic device applications. In this section, we review the stimulated radiation and trapping of polaritons, programmable polaritons, and polariton LEDs in optical cavities strongly coupled with atomically thin TMDs.

### Stimulated relaxed/scattering polaritons

**Fig. 5:**
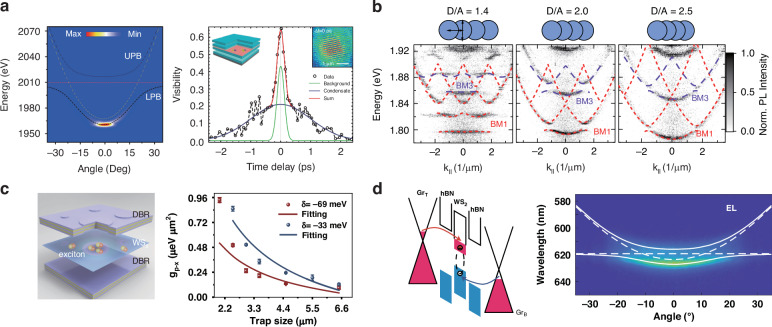
**Polaritonic device applications.** **a** The room temperature polariton condensation in a WS_2_-DBR microcavity. The left panel is the above-threshold angle-resolved photoluminescence map and the right panel shows the coherence visibility of concentrated polaritons as a function of time delay. **b** The angle-resolved photoluminescence map from one-dimensional polariton lattices with different nearest-neighbor coupling strengths. The ratio of diameter D to center-to-center distance A represents the nearest-neighbor coupling strength. **c** Controllable nonlinearity of polaritons in monolayer WS_2_ mesa cavities. The left panel is the schematic of monolayer WS_2_ embedded in two DBRs with cylindrical mesa structures. The right panel shows the estimated polariton-exciton interaction strength versus trap size at the detuning of −33 meV (blue) and −69 meV (red). **d** The room temperature polariton LED is based on monolayer WS_2_. The left panel is the band diagram at a high bias above the threshold, and the right panel shows the angle-resolved electroluminescence (EL) at the current injection of $0{{\rm{.10}} {{\upmu }}{\rm{A}}{{\upmu }}{\rm{m}}}^{-2}$. **a** Reprinted with permission from ref. ^43^ [American Chemical Society]. **b** Reprinted with permission from ref. ^39^ [Springer Nature Limited]. **c** Reprinted with permission from ref. ^114^ [Springer Nature Limited]. **d** Reprinted with permission from ref. ^38^ [Springer Nature Limited]

As bosonic quasiparticles, polaritons can massively occupy a single quantum state triggered by the stimulated scattering, manifesting a nonequilibrium condensation phenomenon and leading to the spontaneous coherence of light emission^108^. Different from conventional photonic lasers, polariton lasing does not require population inversion and can take place at a much lower carrier density, which holds great potential for low-threshold coherence light sources. The emergent layered TMDs provide an excellent platform for exploring the nonequilibrium physics at the atomic scale and elevated temperatures^109^, given by the stable and strong exciton resonance. What is more, the intriguing valley pseudospin opens a new avenue for developing novel multifunctional optoelectronic devices. Therefore, substantial endeavors have been devoted to achieving polariton condensation or lasing based on layered materials. In 2018, by collectively coupling the monolayer MoSe_2_ and GaAs quantum well to a Tamm-plasmon resonance, the hybrid polaritons condensation in the van der Waals system was first realized^110^, as confirmed by the dramatic change of emission intensity and the spectral linewidth around the pump threshold. Additionally, due to the bosonic amplification effect under an above-threshold pump, the valley pseudospin of polariton was also partially retained. Then similar spectral evidence of polariton condensation was observed from monolayer MoSe_2_ in an all-dielectric microcavity, as well as a distinct spatial and temporal coherence^111^. Nevertheless, the condensation is always accompanied by certain local dielectric disorders or strains, which result in photonic energy fluctuations. More importantly, the above experimental demonstrations were both implemented at cryogenic temperatures. In 2021, for the first time, the continuous-wave driven room temperature polariton condensation has been realized in a high-quality microcavity (Fig. 5a), which is considered an important landmark in the TMDs region^43^. The ultralow-threshold (about several nW) polariton lasing was evidenced by the linewidth narrowing, threshold-like polariton emission, and unambiguous spatiotemporal interference under ambient conditions. Moreover, the coherent emission showed an obvious linear polarization with the polarization direction depending on the shapes of the surrounded potential well. Since then, the trapping effect on the polariton properties has been intensively studied. In the strong coupling regime, the whole dephasing rate is generally significantly reduced due to the spatially extended wavefunction inherited from the low-dissipation photonic component, which is less susceptible to the disorder or the optical field gradient. The spatial confinement can further suppress the dephasing, especially from the inhomogeneous broadening. As a result, the motional narrowing and the prolonged temporal coherence of polaritons can be observed^41,42^. Interestingly, the polaritons were capable of ballistically transporting throughout the trap sites, where the transport distance is almost determined by the radiative lifetime of polaritons. On the other hand, the accumulation of polaritons in the potential well also accelerated polariton relaxation by enhancing the parametric scattering rate between particles, which not only further contributes to improving both the spatial and temporal coherence, but also modifies the polariton dynamics and final population in the system. For example, by employing artificial optical confinement, the ground state in the discrete band had managed to be preferentially occupied^112^. Furthermore, when the ground state is tuned below the dark state, by tuning the coupling strength or the photon-exciton detuning, the increased stimulated scattering of polaritons would compete with the fast relaxation of carriers to the dark state^113^. Consequently, the quenched radiation can be efficiently circumvented, which implies that polariton trapping is a valid approach for engineering the band structure of semiconductors.

### Programmable polaritons

The combination of exciton-polaritons with well-controlled artificial potential wells has attracted remarkable interest in the recent few years. Due to its flexibility, polariton potential engineering is a promising candidate not only to emulate complex Hamiltonians but also for the prototype polaritonic device relying on strong nonlinearities and tailored confinement. Considering the small Bohr radius of excitons, engineering photonic potential, for instance, through the photonic lattice and single mesa potential, can be more practical and convenient. In 2021, Lackner et al. revealed strong photon-exciton coupling between artificial photonic lattices and a WS_2_ monolayer in an open cavity at room temperature, as evidenced by the canonical gapped spectrum of Bloch-polaritons^39^. Furthermore, by controlling the nearest-neighbor coupling strength (represented by D/A) in the lattice (as shown in Fig. 5b), they realized the deterministic manipulation of the spatial confinement pattern of TMD polaritons. However, since the detuning between the exciton and cavity photon was up to 180 meV, all of their experiments were carried out far away from the nonlinear regime, which greatly hindered the application of functional polariton-based devices. To confine and manipulate the exciton-polariton in the nonlinear regime at ambient conditions, Luo et al. developed the artificial mesa cavities (Fig. 5c) by patterning the PMMA spacer layer using e-beam lithography^114^. The detuning of their mesa cavities is down to 33 meV, ensuring accessible nonlinear interaction strength due to the large excitonic faction in polaritons. Through improving the spatial confinement strength, the polariton nonlinearity can be increased by at least six times, achieving the combination of strong nonlinearity together with the thermal stability of the TMD polaritons. Additionally, a functional polariton device incorporated with the TMD system has also been reported recently by Zheng et al.^114^ in a planar microcavity etched in the honeycomb structure. By combining the spin-orbit coupling with the pseudo magnetic field induced by the optical stark effect, they realized an all-optically controllable polariton topological insulator. Consequently, all of these progresses pave the way towards developing realistic polaritonic devices and applications based on the TMD MCs.

### Polariton LEDs

Although optical pumping provides an efficient way to investigate fascinating polaritonic phenomena, electrical pumping is critical to push the polaritonic devices into practical applications. The realization of electrically pumped polaritonic devices began with an LED device utilizing a GaAs quantum well structure at 235 K^44^. Subsequently, electrically driven polariton lasers were realized in similar structures^115^. Thanks to the growingly versatile candidates in the strong coupling regime, room temperature polariton LEDs have been developed in organic materials^116^ as well as in inorganic semiconductors^117,118^. However, for 2D TMDs, due to the reduction in the cavity quality factor and Rabi splitting attributed to the loss from graphene electrodes, monolayer microcavity was found to be difficult to meet the strong coupling criteria at room temperature^38^. Implementing a multi-quantum well structure configuration results in a notable enhancement of coupling strength^49^, which can compensate for the decrease in Rabi splitting attributed to electrode loss. In this arrangement, electroluminescence (EL) of exciton-polaritons was observed^38^ (Fig. 5d), leading to the achievement of an LED device with an external quantum efficiency of 0.1%.

## Summary and outlook

As we have observed, TMD materials have been experimentally demonstrated as promising platforms for enriching polaritonic physics and relevant applications at room temperature. In this review, we have summarized recent progress in the study of exciton-polaritons within TMD materials, encompassing various optical cavities, the fundamental properties of TMD polaritons, and TMD-based polaritonic device applications. Although the progress made in the past decade is impressive, there remain tremendous opportunities and challenges in this field, particularly to push polaritonic devices operating at room temperature into reality. In the following, we will elaborate our views and opinions in a perspective manner.

### Nonlinear polaritonic simulators

Photonic simulators, employing photon-based lattices, enable precise exploration of complex Hamiltonians through the manipulation of site arrays with rigorously controlled hopping amplitudes. The combination of nonlinearities and photon-based lattices puts the polariton system at the forefront of research on nonlinear Hamiltonian emulation in the solid state^119^, where the accessibility of polaritons lattices at the micron scale allows to reproduce the physics of less accessible ones. The state-of-the-art of polariton simulators is based on a mapping of the phases of polariton condensates to classical spins^120^. Polariton condensates at connected sites form a graph corresponding to an XY spin system. Assuming that the intensity of polaritons is identical at each site, the state of highest gain in which the collective condensate forms matches the ground state of the corresponding XY spin system. In this way, the polariton system effectively solves a hard optimization problem, to which other (NP class) problems, such as the traveling salesman or knapsack problem^121^, can be mapped. Therefore, nonlinear polaritonic simulators have also inspired a variety of experiments and proposals both in traditional platforms like GaAs-based cavity^120^ and emerging materials poised for room temperature operation^122^.

Regarding TMD polaritons, their optical nonlinear response has been increasingly unveiled in both steady-state and transient conditions, revealing a relatively modest nonlinear interaction strength compared to that of other wide-bandgap semiconductors^47,48^. In addition, recent experimental observations have demonstrated tunable exciton-polaritons^39^ and enhanced nonlinear effects^114^ achieved through photon-based potential landscape engineering. These findings have paved the way for the development of nonlinear polaritonic lattice within TMD-based MCs, enabling the exploration of emerging phenomena, including various topological phases^123–125^, all-optical switching^16,126^, machine learning of phase transitions^127^, and fractional quantum Hall physics^128^. What’s more, it can be anticipated that TMD materials will be likely to appear as potential candidates for nonlinear polaritonic simulators, followed by the advancement in nonlinear polaritonic lattices.

### Quantum exciton-polaritons

Polaritons possess discrete variables such as spin and particle number, alongside continuous variables like amplitude and phase, which can be harnessed as discrete qubits for information encoding, holding important roles in quantum applications^129^. Many theoretical endeavors have been dedicated to modeling exciton-polariton systems, with the aim of realizing fundamental quantum phenomena, including phase transitions^130,131^, quantum quenches^132^, quantum metastability^133^, and the interaction of polaritons with quantum light^134^. In this context, the choice of suitable material platforms plays an important role in harnessing the potential of quantum polaritons. Recent advancements in the fundamental physics of TMD polaritons, particularly their nonlinear effects, have spurred a plethora of proposals for applications in the regime of quantum information. Developments in materials science have enabled the enhancement of interaction strengths by orders of magnitude. Theoretical suggestions that further amplification can be mediated by light are within reach^135^. Taking these advancements, the achievement of polariton quantum blockade appears to be a feasible experimental milestone. Furthermore, recent progress in TMD polaritons, including insights into their valley properties and the effective control of light-matter coupling under the influence of electronic and magnetic fields, provides promise for information processing in polariton simulators and computers. These developments are highly encouraging for applications in information processing and quantum technologies.

### Moiré exciton-polaritons

Besides graphene, another significant material to construct Moiré superlattice is the two-dimensional TMD semiconductors due to the similar lattice constants of the TMD family^136–138^. Recently, many outstanding discoveries have been made in TMD-based Moiré systems, such as twist-angle-dependent exciton dynamics and diffusion^139^, Mott insulating state^140^, generalized Wigner crystal states^141^, the magnetic interaction between holes localized by the Moiré potential^142^, and so on. Therefore, the Moiré excitons strongly coupled with optical cavities are expected to provide an ideal platform to study many-body quantum phenomena, considering their strong interactions, out-of-plane electrostatic dipoles within localized interlayer exciton, and tunable spatial confinement^137^. In the experiment, Förg et al. first integrated a CVD-grown MoSe_2_-WSe_2_ heterobilayer^143^ but suffered from the weak exciton-photon coupling strength due to the small oscillator strength of Moiré excitons. In order to address current problems, Zhang et al. incorporated a MoSe_2_/WS_2_ heterostructure into a planar microcavity and realized the strong light-matter interaction^40^. In theory, there also have been some new physical phenomena proposed based on Moiré systems in the strong coupling regime, such as topological transport^144^ and twist-angle-dependent polariton energy^145^. The flexible tunability inherent in moiré exciton polaritons presents a compelling prospect for delving into and engineering innovative strongly correlated phases of interacting photons. Despite some progress in this field, advancements have been impeded by the challenge of operating at room temperature. Hence, exploring new approaches that allow for the controllability of the moiré potential depth and profile is considered a plausible solution to address this challenge, probably by using the surface potential of a twisted hBN substrate^146,147^.

### Anisotropic exciton-polaritons

Until now, significant advancements in strong light-matter coupling within van der Waals materials have primarily focused on isotropic excitons, exemplified by WS_2_, MoS_2_, WSe_2_, and MoSe_2_. Nevertheless, the investigation of exciton-polaritons is progressively expanding to encompass materials displaying in-plane optical anisotropy. This fascination arises from the facile adjustment of optoelectronic properties by varying crystal thickness, the polarization of the excitation pump, external magnetic and electric fields^148^, as well as sample temperature. In various anisotropic materials, 2D magnetic semiconductors have garnered significant attention due to their novel magneto-optical responses. Since 2D magnetism was discovered in 2017^149,150^, 2D excitons in antiferromagnetic (AFM) semiconductors have revealed intriguing magneto-correlated properties that hold promise for polaritonic research. As a typical example, CrSBr (with a Neel temperature *T*_N_ ~ 132 K) exhibits ferromagnetic (FM) order in monolayer but interlayer AFM coupling along their stacking direction. Under an external magnetic field, the AFM phase of CrSBr can transition into FM phase^151^ with distinct direct-gap band structures and strong anisotropy. As a result, the excitons are found to be spin-correlated with strong anisotropy, high quantum yield, and giant oscillator strength. Of particular interest are the diverse magnons present in CrSBr, which can couple directly with excitons^152^. This example presents an intriguing magneto-optical platform for studying exciton polaritons. Another example is NiPS_3_ (*T*_N_ ~ 155 K), where FM nickel atomic chains could coexist with AFM orders at each layer. Notably, NiPS_3_ introduces a new type of exciton, known as Zhang-Rice singlets, due to spin-orbital entanglement. Unlike conventional excitons formed by Bloch states, these newly formed excitons are many-body correlated states^153^. Moreover, NiPS_3_ exhibits an extremely narrow photoluminescence linewidth (typically <1 meV) compared to 2D TMDs and strong anisotropy^153–155^ like CrSBr. The study of strong coupling in 2D AFM semiconductors is still at its initial stage. Recent advancements have predominantly focused on the realization of the strong coupling regime, and exploring fundamental properties, such as nonlinear polaritonic interactions and behavior of magnon-coupled exciton polaritons^156–159^. Thus, AFM semiconductors hold potential as excellent candidates for exciton-polariton studies, leveraging their giant oscillator strength, strong anisotropy, and magneto-optical control. Additionally, beyond AFM semiconductors, group VII TMDCs such as ReS_2_ and ReSe_2_, which inherit a distorted single-layer trigonal (1 T′) structure of triclinic symmetry, exhibit pronounced in-plane anisotropic optical properties^160,161^. In 2020, Rahul Gogna et al. first reported the self-hybridized, polarized polaritons in ReS_2_ flakes placed on a gold mirror due to its exceptionally high refractive index and anisotropic excitons^162^. However, the absence of Rabi splitting with clear anti-crossing hinders further investigation in ReS_2_. Recent experiments have successfully observed anisotropic exciton-polaritons^163^ with Rabi splitting up to ~68 meV and Rydberg polaritons in ReS_2_^164^. Although recent developments still rely on low temperatures, it is foreseeable that, with the recent rapid progress in nanotechnology and materials science, anisotropic polaritons capable of surviving at room temperature will emerge. Building on these breakthroughs, anisotropic polaritons are poised to create new possibilities, bringing polarization-tunable polaritonic devices operating under ambient conditions closer to fruition.
